# Polyphyllin I Effects *Candida albicans* via Inhibition of Virulence Factors

**DOI:** 10.1155/2023/5645500

**Published:** 2023-01-23

**Authors:** Ai-Mei Sun, Ying-Xian Wang, Guo-Xian Hu, Li Li, Rui-Rui Wang

**Affiliations:** School of Chinese Materia Medica, Yunnan University of Chinese Medicine, Kunming 650500, China

## Abstract

*Paris polyphylla* is often used in Chinese medicine to treat conditions such as carbuncles, trauma, snake bites, and mosquito bites. In the present study, we investigated the effect and mechanism of the morphological transition and extracellular phospholipase activity of *Candida albicans* treated with polyphyllin I (PPI). First, the minimum inhibitory concentration and antifungal activity of PPI were evaluated using the multiple microdilution method and time-killing assays. Then, the effect of PPI on the morphological transition of *Candida albicans* in Spider liquid medium and Sabouraud-dextrose liquid medium containing 10% fetal bovine serum was observed under an inverted microscope and by scanning electron microscopy. Finally, egg yolk agar plates were used to evaluate extracellular phospholipase activity. Gene expression was detected by real-time quantitative polymerase chain reaction analysis. Our results suggest that PPI inhibited the transition from the yeast to the hyphal stage and decreased secreted aspartyl proteinase activity. We further confirmed that PPI significantly downregulated the expression of extracellular phospholipase genes and cAMP-PKA signaling pathway-related genes. Taken together, our results suggest that PPI exerts anti-*Candida albicans* activity by inhibiting virulence characteristics, including the yeast-to-hyphal transition and the secretion of aspartyl proteases and phospholipases. The study results also indicated that PPI could be a promising therapeutic strategy for *Candida albicans*.

## 1. Introduction

*Candida albicans* (*C. albicans*) is one of the most common opportunistic human fungal pathogens in healthy individuals and causes a wide spectrum of diseases [1, 2], from superficial mucosal infections to life-threatening systemic disorders in immunocompromised human hosts due to virulence factors [3–5], such as protease production and hyphae [6, 7]. As a concurrent infection with other diseases, *C. albicans* is associated with high morbidity, prolonged hospital stays, high relapse rates, and substantial healthcare costs. The recent emergence of COVID-19 in patients with *C. albicans* coinfection has been increasingly described in the literature. Among patients with COVID-19 admitted to the intensive care unit (ICU) in one United States (US) hospital, 8.9% developed candidemia, which resulted in longer ICU stays than in patients with COVID-19 without candidemia, with *C. albicans* being the most common *Candida* infections [8]. *C. albicans* can assume at least three distinct morphologies according to the environmental conditions: yeast-like, pseudohyphal, and true hyphal. It adapts well to different environmental niches. Regardless of the specific niche or site of infection, the ability of *C. albicans* to cause disease has been closely linked to its ability to undergo morphogenic transitions to either pseudohyphal or true hyphal stages [9]. The current therapeutic options for *C. albicans* are highly limited owing to problems of insufficient drug efficacy, severe toxicity, adverse side effects, high cost, and the emergence of drug-resistant strains to clinically available antifungal drugs [10, 11]. Therefore, the discovery of novel antifungal agents and strategies is necessary.

**Figure 1:**
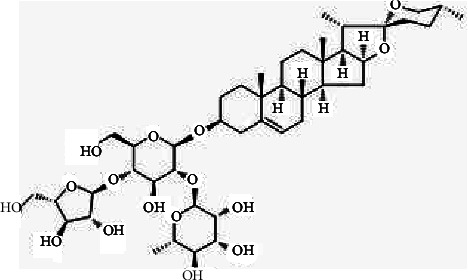
Chemical structure of polyphyllin I (PPI).

Natural products have gained increasing attention for their potential use against fungi [12]. The rhizome of *Paris polyphylla* is called Chong-Lou, and it is a traditional antipyretic detoxicate Chinese medicinal herb, which has been used for a broad range of applications in clinical practice for thousands of years [13]. *Paris polyphylla* has been applied to manage parotitis, mastitis, sore throat, snakebites, convulsion, fractures, abscesses, and various human malignancies [14]. Polyphyllin I (PPI), as illustrated in Figure 1, also called Chong Lou saponin I, a steroidal saponin, is one of the main active ingredients extracted from *Paris polyphylla*. In recent years, researchers have investigated the active ingredients and pharmacological mechanisms of PPI. PPI has many pharmacological effects, such as antitumor [15, 16], anti-inflammatory [17], antibacterial [18, 19], anti-Alzheimer's disease, and immune regulation [13]. PPI is also effective against *C. albicans*, but the antifungal mechanism remains unclear.

## 2. Materials and Methods

### 2.1. Strains, Media, and Reagents

CA23, CA187, CA5408, CA3511, CA800, CA602, CA799, CA558, and CA3816 were generous gifts from Prof. Yu-ye Li, The First Affiliated Hospital of Kunming Medical University (Yunnan, China). The fluconazole (FLC)-sensitive *C. albicans* strain SC5314 was purchased from the American Type Culture Collection (ATCC). ATCC10231 and SC5314 were treated successively with FLC to obtain the FLC-resistant*C. albicans* strains (ATCC10231FR and SC5314FR, respectively). These strains were refreshed from storage at −80°C and subcultured on Sabouraud-dextrose-agar (SDA) at least twice at 37°C, and a single colony was inoculated into Sabouraud-dextrose-broth (SDB) at 37°C for 16–18 h before each experiment to ensure viability. For all experiments, only cultures in the logarithmic growth phase were used. SDB was used for *C. albicans* culture, and Spider liquid medium (1% mannitol, 1% nutrient broth, 0.2% K_2_HPO_4_) and SD + 10% fetal bovine serum (FBS) liquid medium (10 g of peptone and 40 g of dextrose in 1000 mL of ddH_2_O supplemented with 10% FBS) were used for hyphal induction. Polyphyllin I (Chonglou Saponin I, purity ≥ 98%) was purchased from Plant Origin Biological, and FLC was purchased from HelioEast Company (Nanchang, China). All drug solutions were dissolved in dimethyl sulfoxide (DMSO) (50 mg/mL). Stock solutions were stored at −20°C until use.

### 2.2. Antifungal Susceptibility Testing

The MIC_50_ of PPI and FLC against *C. albicans* was determined according to Clinical and Laboratory Standards Institute (CLSI) guidelines [20]. Briefly, PPI and FLC were serially diluted 5-fold to final concentrations of 200-0.064 *μ*g/mL. Then, a volume of 100 microliters of 2 × 10^5^ CFU/mL *C. albicans* (CA23, CA187, CA5408, CA3511, CA800, CA602, CA799, CA558, CA3816, ATCC10231FR, and SC5314FR) were added to 96-well plates. Cell culture wells without drugs were used as experiment controls, and medium-only wells were used as blank controls. The final volume in each well was 200 *μ*L. The plate was then placed at 37°C for 24 hours. Absorbance at 630 nm was measured using a microplate reader. The inhibition ratio of the drugs was calculated as follows: inhibition ratio = [1 − (OD_Treated_ − OD_Blank_)/(OD_Control_ − OD_Blank_)] × 100%. MIC_50_ was calculated using GraphPad Prism 8.0 software. Three biological replicates were performed for each strain, and the experiments were repeated three times.

### 2.3. Time-Killing Curves

CA23 yeast cells were grown until the log phase, diluted to a final concentration of 1 × 10^5^ CFU/mL, and coincubated with PPI (at final concentrations of 1 *μ*g/mL, 2 *μ*g/mL, and 4 *μ*g/mL) or FLC (at a final concentration of 4 *μ*g/mL) in a 37°C shaking incubator. *C. albicans* without drug treatment was used as a control. After 0, 2, 4, 8, 12, 24, 36, 48, and 72 h of incubation, the solution in each group at each time period was placed on a mixing shaker to assure complete mixing. Then, 200 *μ*L of samples were placed in a sterile 96-well plate, and OD_630_ values were determined with a microplate reader. Triplicate wells of each group were assayed, and the experiment was repeated three times. The growth curve was plotted according to the OD_630_ values at each time point.

### 2.4. *C. albicans* Hyphal Morphology Assay

For yeast-to-hyphae transition, yeast cells were induced in Spider liquid medium and SD + 10% FBS liquid medium [21]. A 1 mL suspension of CA23 cells (1 × 10^5^ CFU/mL) treated with FLC (4 *μ*g/mL) or PPI (4 *μ*g/mL, 2 *μ*g/mL, or 1 *μ*g/mL) alone was incubated in 24-well plates at 37°C. At 4 h and 8 h, all wells were observed and photographed using an inverted microscope.

### 2.5. Scanning Electron Microscopy

The morphology of CA23 cells was observed by scanning electron microscopy (SEM) [22]. Briefly, *C. albicans* cells (1 × 10^5^ CFU/mL) were coincubated with PPI or FLC in a 6-well flat-bottomed microplate at 37°C for 8 h, and the cells were collected by centrifugation. The cells were washed three times with sterile phosphate-buffered saline (PBS). The resulting suspension (10 *μ*L) was dropped on sterile slides and allowed to dry. Then, the cells were fixed with 5% glutaraldehyde overnight, and the slides were washed gently three times with PBS. The cells were dehydrated in a series of ethanol solutions (30%, 10 min; 70%, 10 min; 90%, 10 min; and 100%, 10 min) and allowed to air dry. The slide was adhered to a metal plate with carbon tape and placed in a high-vacuum sputter coater for gold plating. After the sample was prepared, it was placed in a scanning electron microscope in the high-vacuum mode at 15 kV for observation and image acquisition.

### 2.6. Effect of Antifungal Agents on Phospholipase Activity

The phospholipase activity of CA23 cells was determined according to a previous method [23]. CA23 cells were harvested in the logarithmic growth phase and diluted to 1 × 10^5^ CFU/mL. A 10 *μ*L sample of the cells (treated and untreated with antifungal agents) was spotted onto the center of egg yolk agar plates containing 1% peptone, 3% glucose, 5.73% NaCl, 0.055% CaCl_2_, and 10% of egg yolk emulsion and incubated for 72 h at 37°C. Three replicate samples were designed for each group. Then, precipitation zones of different sizes were observed around the colonies, and the diameter of the colonies and precipitation zones was measured with digital Vernier calipers. To assess the effect of antifungal agents on phospholipase activity, Pz values were calculated according to the formula: Pz = colony diameter/(colony diameter + precipitation zone). Pz = 1.00 indicated no activity, Pz = 0.90–0.99 indicated weak enzymatic activity, Pz = 0.70–0.89 indicated moderate activity, and Pz ≤ 0.69 indicated strong enzymatic activity [24]. Therefore, higher Pz values indicated lower *C. albicans* phospholipase activity. Experiments were performed in triplicate with at least three independent repetitions, and the data obtained were averaged.

### 2.7. cAMP Rescue Assay

To verify the effect of cAMP on the cAMP-PKA-Efg1 pathway after PPI treatment, *C. albicans* was prepared in cell culture medium (1 × 10^5^ CFU/mL) and coincubated with PPI (final concentration, 2 *μ*g/mL) or FLC (final concentration, 4 *μ*g/mL) in a 24-well plate at 37°C for 4 h and 8 h with or without dibutyryl-cAMP (db-cAMP). The db-cAMP-free group served as the control. Images were acquired using an inverted microscope.

### 2.8. Quantitative Real-Time Polymerase Chain Reaction Analysis

To evaluate the molecular mechanism of PPI, quantitative real-time polymerase chain reaction (qRT-PCR) analysis was performed [25, 26]. CA23 cells (1 × 10^5^ CFU/mL) were coincubated with FLC (final concentration, 4 *μ*g/mL) or PPI (final concentration, 2 *μ*g/mL) with shaking at 37°C for 16 h. A drug-free sample served as the growth control. Fungal cells were harvested by centrifugation at 3500 rpm for 5 min and washed with PBS three times. The fungal cells were ground in liquid nitrogen, and total RNA was extracted using Trizol reagent. RNA concentration and purity were determined using a NanoDrop Lite spectrophotometer and by electrophoresis. Total RNA (1 *μ*g) was reverse-transcribed into cDNA with random primers in a 20 *μ*L reaction volume using a GoScript Reverse Transcription Kit following the manufacturer's instructions. After cDNA was synthesized, the expression levels of secreted aspartyl proteinase-related genes (*SAP1*, *SAP2*, *SAP3*, and *SAP4*) and phospholipase B1, phospholipase B2, and cAMP-PKA signaling pathway-related genes (*GPR1*, *GPA2*, *CYR1*, *TPK1*, *EFG1*, *ECE1*, and *HWP1*) were assessed by qRT-PCR. qRT-PCR mixtures (20 *μ*L) containing cDNA, GoTaqR qPCR Master Mix, sterile nuclease-free water, and gene primers were freshly prepared. The primer sequences used for the amplification of specific genes are shown in Table 1. Quantitative PCR reactions were performed using a Lightcycler® 96 fluorescence quantitative PCR system (Roche) with the following cycles: 95°C for 60 s for predenaturation, then 95°C for 15 s, annealing at 55°C for 30 s, and extension at 72°C for 30 s for a total of 40 cycles. The signal from each sample was normalized to *ACT1* gene expression. Relative quantitation analysis of the gene expression data was conducted according to the 2^−(ΔΔCt)^ method. Independent experiments were repeated three times with similar results. The data are from one representative experiment.

### 2.9. Cytotoxicity

Drug cytotoxicity tests were evaluated using the MTS (3-(4,5-dimethylthiazol-2-yl)-5-(3-carboxymethoxyphenyl)-2-(4-sulfophenyl)-2H-tetrazolium) [27, 28]. Human bronchial epithelial cells (16HBE) were obtained from the American Type Culture Collection. They were cultured in Roswell Park Memorial Institute (RPMI) 1640 medium (Gibco) supplemented with 10% FBS and 1% penicillin-streptomycin. 16HBE cells were seeded into 96-well culture plates at a density of 1 × 10^4^ cells per well and incubated for 24 h at 37°C and 5% CO_2_. The cell culture supernatant was aspirated, and cells were washed and then treated with FLC (4 *μ*g/mL), PPI (2 *μ*g/mL), or DMSO for 24 h. Cell culture wells without drug supplementation were used as the experiment controls, and medium-only served as the blank controls. The cells were further incubated for 24 h before determining cell viability by the MTS assay according to the manufacturer's instructions. Then, the OD values of each well were measured with a microplate reader at 492 nm. The percentage of growth inhibition was calculated as follows: growth inhibition (%) = [1 − (OD_experiment_ − OD_Blank_)/(OD_control_ − OD_Blank_)] × 100% [29].

### 2.10. Statistical Analysis

All statistical analyses were performed using Prism 8 (GraphPad) software. All experiments were independently repeated at least three times. The data are expressed as the means ± SD of triplicate experiments and differences between groups were evaluated by ANOVA. *P* values of <0.05 was considered statistically significant.

## 3. Results

### 3.1. Antifungal Susceptibility Testing

The results are shown in Table 2. PPI exhibited potent antifungal activity against CA23, CA187, CA5408, CA3511, CA800, CA602, 10231FR, and SC5314FR, with MIC_50_ values of 0.90–47.30 *μ*g/mL, whereas the MIC_50_ for FLC of all the tested strains was >200 *μ*g/mL, indicating no antifungal activity. No antifungal effects on the *C. albicans* clinical strains (CA799, CA558, and CA3816) were observed by treatment with PPI. All data are the average of triplicate experiments. The MIC_50_ values of the 11 strains were similar, as was the CA23 strain compared to the other 10 strains. CA23 is more stable and easy to culture, so CA23 was selected as the research strain.

### 3.2. Time-Killing Curves

**Figure 2:**
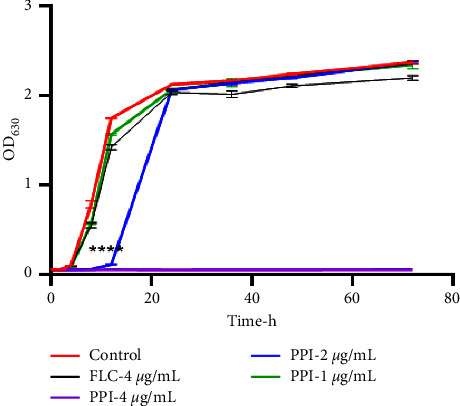
Growth curve of *C. albicans* strain CA23 treated with PPI. *C. albicans* cells were incubated with different doses of PPI at 37°C with shaking. The OD_630_ of each group was detected at specific time points using a microplate reader. The results are shown as means ± standard deviation (SD). The PPI groups were compared to the growth control group. ^*∗∗∗∗*^*P* < 0.0001.

The OD_630nm_ values of *C. albicans* cultures containing FLC (4 *μ*g/mL) and PPI (1, 2, and 4 *μ*g/mL) were determined to assess the dynamic antifungal effect of PPI on the growth of *C. albicans*. The results are shown in Figure 2. After 4 h of PPI treatment, there was no significant difference compared to the control group. After 8 h and 12 h of PPI treatment (2 and 4 *μ*g/mL), *C. albicans* growth was significantly inhibited (*P* < 0.0001). Twelve hours later, *C. albicans* treated with PPI (2 *μ*g/mL) grew rapidly and then slowed before reaching a plateau at 24 h. The PPI (4 *μ*g/mL) group exhibited fungicidal activity. Our results showed that PPI dose-dependently inhibited the growth of *C. albicans*. The OD values were not significantly different between the FLC group and the control group. OD values were measured at different time points of growth and shown on the *Y*-axis and time on the *X*-axis in Figure 2. The curves represent the trends in *C. albicans* growth. The results demonstrated that PPI could actively inhibit *C. albicans*, which is consistent with the microdilution assay results.

### 3.3. *C. albicans* Hyphal Morphology Assay

**Figure 3:**
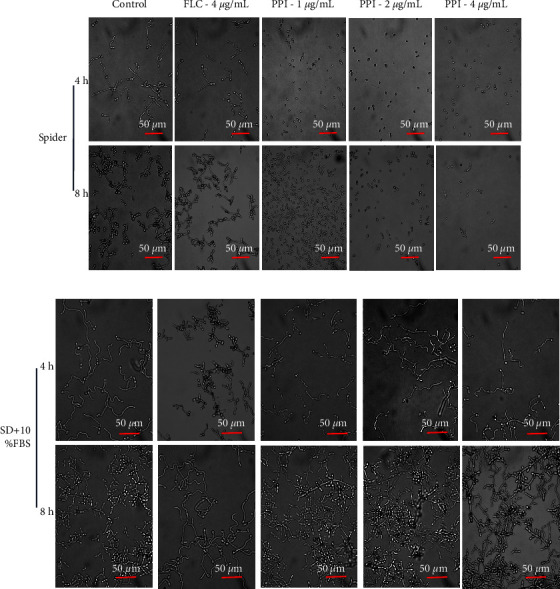
Effect of PPI and FLC on the yeast-to-hyphal transition of *C. albicans* in two different hyphae-inducing media. (a) Effect of PPI or FLC on CA23 hyphal growth induced by Spider medium at 37°C without shaking for 4 h and 8 h. PPI was diluted in Spider medium to final concentrations of 1, 2, and 4 *μ*g/mL. FLC was diluted in Spider medium to a final concentrations of 4 *μ*g/mL. The untreated strain in medium-only was set as the control. Images were photographed at ×40 magnification. (b) Effect of PPI or FLC on CA23 hyphal growth induced by SD + 10% FBS medium at 37°C without shaking for 4 h and 8 h. Treatments: control group (no treatment, medium-only), FLC group (4 *μ*g/mL), and PPI group (1, 2, and 4 *μ*g/mL). Images were photographed at ×40 magnification. Three independent experiments were performed.

The effect of PPI on the hyphal formation of *C. albicans* in Spider liquid medium (Figure 3(a)) and SD + 10% FBS medium (Figure 3(b)) was observed using an inverted microscope. The control group formed hyphae at 4 h. The control group formed dense and long hyphae in Spider medium at 8 h. The FLC treatment did not significantly inhibit the hyphal formation of *C. albicans* compared to the control group at any time. The hyphae growth of *C. albicans* was effectively inhibited after PPI treatment; only yeast-like cells appeared but no obvious hyphae. This result suggests that PPI could effectively inhibit the transition of yeast to hyphae in Spider liquid medium. Interestingly, we found that PPI and FLC did not inhibit serum-induced *C. albicans* hyphae growth at any time.

### 3.4. Scanning Electron Microscopy

**Figure 4:**
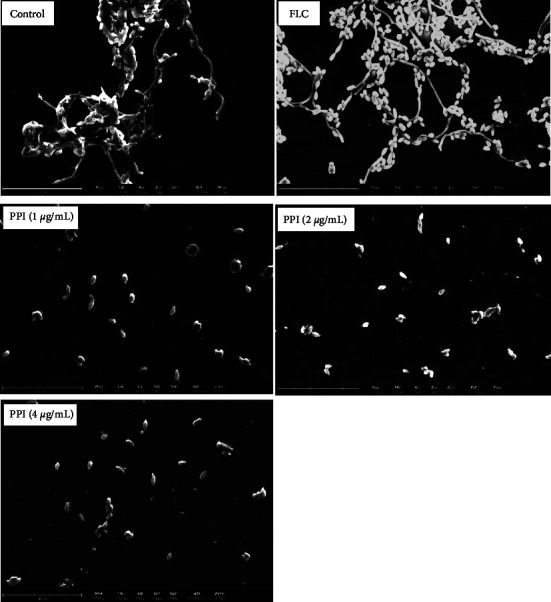
Effect of PPI or FLC on the morphological changes of *C. albicans* in Spider medium. Morphology was observed by SEM (3000x) after incubation at 37°C for 8 h treatments: control group (no treatment, medium-only), FLC group (4 *μ*g/mL), and PPI group (1, 2, and 4 *μ*g/mL).

To further confirm the antifungal activity of PPI, the morphological appearance of *C. albicans* was examined by scanning electron microscopy. SEM images (Figure 4) of the untreated control group showed mixtures of pseudohyphae, crisscrossing hyphae, and few yeast cells. Compared to the untreated control, FLC reduced the length of *C. albicans* hyphae and increased the number of yeast-like cells. *C. albicans* exposed to 1, 2, and 4 *μ*g/mL PPI only showed yeast cells. Our results show that PPI inhibits the transition from yeast to hyphae.

### 3.5. Effect of Antifungal Agents on Phospholipase Activity

Lower Pz values indicate higher phospholipid activity. Therefore, the more enzyme that is produced, the lower the Pz value. FLC showed inhibitory effects on CA23 phospholipase activity (Table 3). Phospholipase activity was inhibited by PPI, as the Pz value was 0.85, compared to the very strong activity of the control sample with a Pz value of 0.67. These results indicated that PPI decreased the virulence of *C. albicans* by repressing phospholipase activity, thereby exerting antifungal effects.

### 3.6. cAMP Rescue Assay

**Figure 5:**
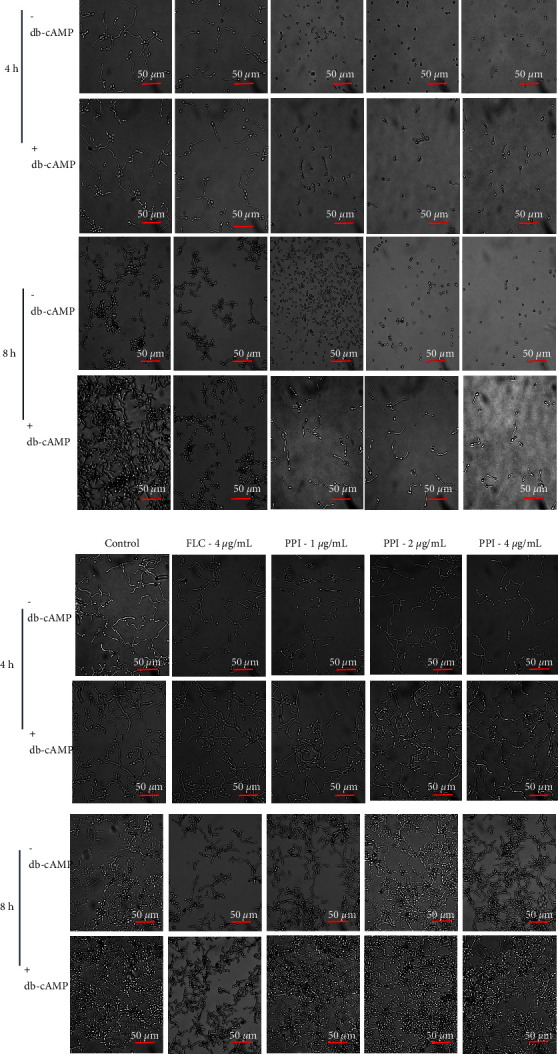
Exogenous cAMP restores the hyphal formation of *C *. *albicans*, which is inhibited by PPI. Images were photographed (40x magnification) after incubation in two different hyphae-inducing media at 37°C for 4 h and 8 h. (a) Exogenous cAMP restored PPI-inhibited hyphal formation in Spider medium with or without db-cAMP for 4 h and 8 h. (b) Exogenous cAMP restored PPI-inhibited hyphal formation in SD + 10% FBS medium with or without db-cAMP for 4 h and 8 h.

We confirmed that the addition of exogenous db-cAMP rescued the inhibitory effect of PPI on hyphal formation in Spider liquid medium and restored the ability of *C. albicans* to form hyphae (Figure 5(a)). Thus, PPI could reduce cAMP levels in fungal cells in Spider medium. However, it is interesting to note that PPI did not affect *C. albicans* hyphal formation in SD + 10% FBS medium (Figure 5(b)) before or after the addition of exogenous cAMP.

### 3.7. Quantitative Real-Time qPCR

**Figure 6:**
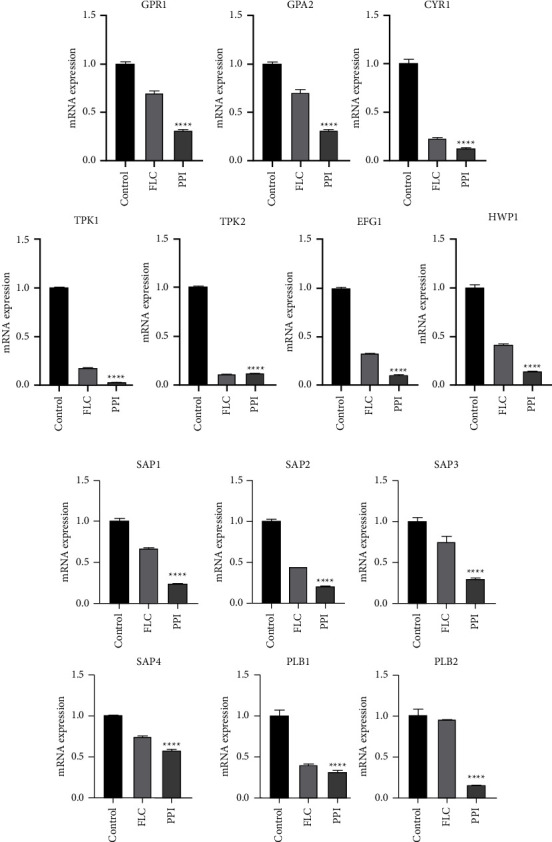
Changes in the expression of cAMP-PKApathway-related genes and hydrolase-related genes in the CA23 strain by PPI treatment. (a) The expression levels of Ras1-cAMP-Efg1 pathway-related genes *GPR1*, *GPA2*, *CYR1*, *TPK1*, *TPK2*, *EFG1*, and *HWP1*. (b) The expression levels of protease-encoding genes *SAP1*, *SAP2*, *SAP3*, and *SAP4*, and phospholipase-encoding genes *PLB1* and *PLB2*. Gene expression was detected by qRT-PCR. The untreated strain in medium-only was used as the control. *ACT1* was used as the internal reference gene and quantified using the 2^−ΔΔCT^ method. ^*∗∗∗∗*^*P* < 0.0001 compared to the control group.

The mechanism of filamentation inhibition by PPI was further investigated, and qRT-PCR was conducted to explore the effect of PPI on the cyclic adenosine monophosphate-protein kinase A (cAMP-PKA) pathway-related genes: *GPR1*, *GPA2*, *CYR1*, *TPK1*, enhanced filamentation growth factor-1 (*EFG1*), cell elongation protein 1 (*ECE1*), hyphal wall protein-1 (*HWP1*), and aspartyl proteases-encoding-related and phospholipase-encoding-related genes. Regarding gene expression changes in *C. albicans* (Figure 6), cAMP-PKApathway-related genes (*GPR1*, *GPA2*, *CYR1*, *TPK1*, *EFG1*, *ECE1*, and *HWP1*) were significantly downregulated after PPI treatment compared to the control group. The expression of the protease-encoding secreted aspartyl protease *SAP1*, *SAP2*, *SAP3*, and *SAP4* genes, as well as the phospholipase-encoding gene phospholipase *B1* (*PLB1*) and *PLB2*, were strongly inhibited by PPI.

### 3.8. Cytotoxicity

**Figure 7:**
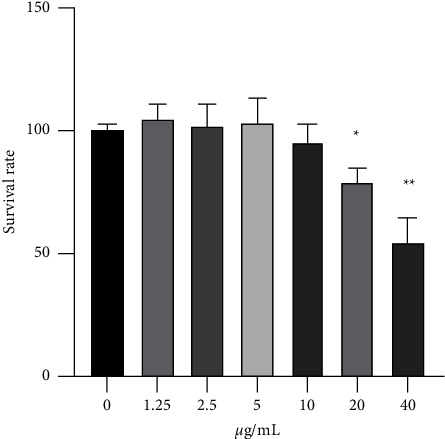
Effect of PPI on the cell survival rate of 16HBE cells. 16HBE cells were incubated with different concentrations of PPI (1.25, 2.5, 5, 10, 20, and 40 *μ*g/mL) for 24 h, and the cell survival rate was measured by the MTS assay kit. All data are presented as mean ± SD. ^*∗*^indicates statistical significance between control and PPI-treated groups.

To assess cell viability following the treatments, cytotoxicity was analyzed by the MTS assay. PPI exerted inhibitory effects on the proliferation of 16HBE cells at high concentrations. At concentrations ≤10 *μ*g/mL, the toxicity of PPI in 16HBE cells was low, at about 79.26% viability compared to the control groups (Table 4, Figure 7).

The data in Table 4 represent the mean ± SD. ^*∗*^indicates statistical significance between the control and PPI-treated groups. The blank group contained cell-free medium. The viability of control cells cultured in pure medium was 100%. The drug control group was a drug-containing medium without cells. In the drug-treated group, cells were treated with different concentrations of PPI for 24 h, and the cell survival rate was determined by the MTS assay.

## 4. Discussion and Conclusion

*C. albicans* is a dimorphic commensal fungus commonly present in the healthy microbiota of the oral, gut, and vaginal mucosae. It can become pathogenic when the balance between the fungus, mucosa, and host defense mechanisms is interrupted, leading to the emergence of candidiasis [30, 31]. Treating *C. albicans* is challenging due to its close evolutionary relationship with the human host, the limited efficacy and side effects of antifungal drugs, and the emergence of drug-resistant strains. It poses a threat not only to global health but also to the economy. New antifungal agents that are effective against fungal pathogens are urgently needed [32–34].

The generation of filamentous hyphae is a defining feature of *C. albicans* pathogenesis [1, 35]. The transition, which depends on its environment, is important for *C. albicans* infection, colonization, and the evasion of the host immune system. Yeast-form cells facilitate dissemination through the bloodstream, while hyphal cells cause tissue damage and invasion, as well as aid in the escape of phagocytic cells [36]. The cAMP-PKA pathway in *C. albicans* plays a role in yeast-to-hyphal transition [37]. In the cAMP-PKA pathway, *GPR1* (encoding the G protein-coupled receptor Gpr1) acts through the G-protein Gpa2 on the cAMP pathway in response to environmental stimuli. The deletion of *CaGPR1* causes strong defects in the yeast-to-hyphal transition on various solid hypha-inducing media [38]. *C. albicans* expresses a single adenylyl cyclase, which is encoded by *CYR1* (also known as *CDC35*) and can be activated by the G protein Gpa2 to form cAMP [37]. The formation of hyphae is defective in cyr1Δ mutants [39]. cAMP acts as a key second messenger, and the binding of Bcy1 by cAMP activates PKA isoforms thiamin pyrophosphokinase 1 (Tpk1) and Tpk2 to activate Efg1 and other PKA targets. *TPK1* and *TPK2* encode both isoforms of PKA catalytic subunits, and different cAMP-dependent pathways determine the cellular activity of the catalytic subunits depending on the nature of the inducing medium [40]. Tpk1p is required for the derepression of certain amino acid biosynthetic genes and plays a positive role in the morphogenetic process. Studies showed that the TPK1Δ strain exhibited a delayed morphogenetic shift in several liquid-inducing media compared to the CAI4Δ and TPK2Δ strains [41]. The EFG1 gene, which encodes for enhanced filamentous growth protein 1 (Efg1), is a transcription factor that regulates filamentation, metabolism, biofilm formation, and virulence [42, 43]. Hyphal wall protein 1 (*HWP1*) and candidalysin (*ECE1*) are the core filamentation genes and are highly expressed in *C. albicans* hyphae [44]. Hyphal elongation or yeast-to-hyphal transition has been shown to contribute to fungal invasion, which is mediated by the cell elongation 1 gene (*ECE1*), or by commensalism, which is mediated by Efg1p during Candida mucosal infections [31]. Efg1 is responsible for the positive regulation of the expression of several hyphal-specific genes, including *SAP3-6*, *ALS3*, and *HWP1*. Mutants lacking Efg1 significantly decreased the expression of *SAP3* and *SAP5*, and the virulence of the strain decreased [45–47]. *HWP1*, *ECE1*, and *SAP3* were regulated by Efg1. To sum up, hyphal growth is very important for *C. albicans* host invasion, and the molecular mechanism is relatively clear. Thus, the prevention of hyphal formation is considered an effective treatment option.

This study demonstrated that PPI markedly reduced the hyphal formation of *C. albicans* at a concentration of 2 *μ*g/mL. The microscopic analysis demonstrated that PPI effectively inhibited hyphal development in a dose-dependent manner. Gene expression analysis revealed that PPI significantly downregulated the expression of hydrolase-related genes and cAMP-PKApathway-related genes, consistent with the phenotypic analysis. The expression of *GPR1*, *GPA2*, *CYR1*, *EFG1*, *AlS3*, *ECE1*, and *HWP1* was significantly downregulated by PPI treatment. For further verification, *C. albicans* treated with PPI was also treated with db-cAMP, a functional analog of cAMP, to evaluate whether it could restore hyphal growth function in culture. db-cAMP partially restored the hyphae-forming ability of *C. albicans*, consistent with previous studies. Our results suggest that PPI exerts its anti-*C. albicans* effect mainly by inhibiting the cAMP-PKA pathway. Hyphae also express specific virulence factors, such as degradative enzymes (the Sap family of secreted aspartyl proteases), cell surface adhesins (adhesin agglutinin-like protein 3 (Als3) and Hwp1, and the pore-forming toxin candidalysin (Ece1). During the invasion, pathogenic fungi secrete various hydrolytic enzymes to facilitate host entry. Such secreted fungal enzymes are divided into three major groups: secreted aspartyl proteases, lipases, and phospholipases. Secreted hydrolytic enzymes disrupt host cell membranes, promote cell adhesion and biofilm formation, impair host barrier function, and damage host tissues [48]. Hydrolytic enzymes, aspartyl proteases, and phospholipases secreted by *C. albicans* are the best characterized [49, 50]. Secreted aspartyl proteinase (Sap) is an extracellular protease secreted by *C. albicans*. In human mucosal diseases, it is responsible for adhesion and invasion [51]. The transition from round budding cells to long hyphal forms and the production of Saps are considered the virulence-associated factors of *C. albicans*. Saps are the products of a family of 10 SAP genes divided into subfamilies based on amino acid sequence homology alignment (*SAP1* to *SAP3*, *SAP4* to *SAP6*, *SAP9*, and *SAP10*) [52, 53]. *SAP1*, *SAP2*, and *SAP3* contribute to the overall virulence of *C. albicans* and presumably play an important role in the process of disseminated infection. *SAP4*, *SAP5*, and *SAP6* form a group distinct from *SAP1*, *SAP2*, and *SAP3*. When guinea pigs and mice were injected intravenously with the delta saps 4, 5, and 6, triple-homozygous null mutant DSY459, their survival time was significantly longer than that of control animals infected with wild-type SC5314 [47, 54]. Saps 1–6 are required for invasive disease. In guinea pig and murine models of invasive disease, deletions in Sap1–6 attenuated virulence [47]. The SAP family genes encoding proteins Sap1, Sap2, Sap3, and Sap4 are required for hyphal formation and maintenance. Gene expression analysis revealed that PPI significantly downregulated the expression of Sap1, Sap2, Sap3, and Sap4, indicating that PPI could decrease the production of Saps and thus the virulence of the strain.

*C. albicans* phospholipase is an important virulence factor. In recent years, phospholipase has been increasingly reported [55]. Phospholipases are divided into four major classes, A, B, C, and D, according to the specific bond targeted in the phospholipid molecule. Phospholipase B mainly hydrolyzes lysophospholipid bonds [56]. The relative risk of death was 5.6-fold higher in mice infected with higher-phospholipase-secreting strains than with the low-phospholipase secretors [57]. Phospholipase B contributes to the pathogenicity of *C. albicans* by traversing host cell membranes, a process that may increase the rate of infection dissemination [58]. *PLB1*, which codes for phospholipase B/lysophospholipase in yeast, was required for virulence in an animal model of candidiasis; a gene-deleted strain produced less phospholipase *in vitro* and was less virulent than the wild-type [56, 59]. The study found that sodium houttuynate inhibited the relative expression of *PLB1* and *PLB2*, thus exerting anti-*C. albicans* activity [60]. Our results also showed that PPI could decrease the relative expression of *PLB1* and *PLB2*, inhibit the secretion of phospholipase by *C. albicans*, and reduce the virulence of the strain.

In summary, PPI converted the pathogenic hyphal form to the less-virulent yeast state of *C. albicans* by inhibiting the expression of hyphae-related virulence factors. Thus, PPI can be used not only for the prevention of *C. albicans* infections but also as an effective therapeutic agent.

## Figures and Tables

**Table 1:** Primer sequences for qRT-PCR reactions.

Oligo Name	Sequence (5′to 3′)	Length (bp)
*GPR1*-forward	TTCATCTCGCCAGCAACAGT	178
*GPR1*-reverse	ATTACATTTCGGTGGGGGCT	

*GPA2*-forward	CCACCACCAAAACAACGCAA	148
*GPA2*-reverse	CTTTCACTTCAGGGGTCTCGT	

*CYR1*-forward	ACTTGGTGACTGCAGACTGG	110
*CYR1*-reverse	ACCCATACGAACCGACAACC	

*TPK1*-forward	GCTGCCGAAGTATTTTTGGCT	194
*TPK1*-reverse	GCCACCACTTCAGGAGCAAT	

*EFG1*-forward	AATGTGGCCCAAATGACACG	131
*EFG1*-reverse	GCCATGGCCAATGCTCTTTC	

*ECE1*-forward	GCCACTGGTGTTCAACAATCC	123
*ECE1*-reverse	AGTTTCCAGGACGCCATCAA	

*HWP1*-forward	CCGGAATCTAGTGCTGTCGT	185
*HWP1*-reverse	GCAGCACCGAAAGTCAATCTC	

*SAP1*-forward	GCTACGCTAACGGTCAACCT	170
*SAP1*-reverse	AGCAGCAATGTTTGAAGCAGA	

*SAP2*-forward	CAATGAAGCCGGTGGTAG	108
*SAP2*-reverse	GTGGCAGCATCTGGAGAA	

*SAP3*-forward	TCAAGCTGGTCAAGGACAAGA	196
*SAP3*-reverse	ATCGGCAAATTGTTGCTTTGTG	

*SAP4*-forward	TGCCGATGGTTCTGTTGC	154
*SAP4*-reverse	CCTGGTGGCTTCGTTGCT	

*PLB1*-forward	CATTCAGTGGCGGAGGGTAT	155
*PLB1*-reverse	TCCAACTAACCACGATCCACC	

*PLB2*-forward	TGGGAGAGCTTTGAGTCACC	154
*PLB2*-reverse	GAGCACAGTGTTTGGTTCCC	

*ACT1*-forward	ACGGTGAAGAAGTTGCTGCT	180
*ACT1*-reverse	TGGATTGGGCTTCATCACCA	

**Table 2:** MIC_50s_ of PPI and FLC against *C. albicans*.

*C. albicans*	MIC_50_ (*μ*g/mL)	
	FLC	PPI
CA23	>200	2.00 ± 0.31
CA187	>200	0.95 ± 0.06
CA5408	>200	6.36 ± 0.94
CA3511	>200	4.37 ± 0.01
CA800	>200	3.26 ± 0.17
CA602	>200	46.00 ± 1.30
CA799	>200	>200
CA558	>200	>200
CA3816	>200	>200
ATCC10231-FR	>200	0.90 ± 0.01
SC5314-FR	>200	0.98 ± 0.01

FLC, fluconazole; PPI, polyphyllin I; CA, *C. albicans*. MIC_50_ was defined as the 50% inhibition of fungal growth compared to control group growth; the data are shown as mean ± SD.

**Table 3:** Effect of PPI on the phospholipase activity of CA23.

Groups	Pz value	Phospholipase activity
Control	0.67 ± 0.02	Strongly
FLC	0.76 ± 0.03	Moderate
PPI	0.85 ± 0.04	Moderate

Each value is the average of three independent experiments ± SD.

**Table 4:** Cell survival rates.

Group	Concentration (*μ*g/mL)	OD values	Survival rate (%)
Blank	—	0.1134 ± 0.0053	—

Drug control	40	0.1340 ± 0.009	—
	20	0.1431 ± 0.0081	—
	10	0.1357 ± 0.0143	—
	5	0.1378 ± 0.0162	—
	2.5	0.1453 ± 0.0197	—
	1.25	0.1403 ± 0.0257	—

Control	0	1.1460 ± 0.0110	100.00

Drug-treated	40	0.5739 ± 0.0754	54.59^*∗∗*^
	20	0.7818 ± 0.0628	79.26^*∗*^
	10	0.9046 ± 0.0554	95.42
	5	0.9658 ± 0.0789	102.76
	2.5	0.9706 ± 0.0573	102.41
	1.25	0.9852 ± 0.0299	104.85

## Data Availability

The data used to support the findings of this study are included in the article.
